# Research on Powder Convergence Characteristics of Powder Feeding Nozzle in Wide-Band Laser Cladding

**DOI:** 10.3390/mi17050515

**Published:** 2026-04-23

**Authors:** Erhao Zhou, Jianjun Peng, Bingjing Guo, Junhua Wang, Xiaojun Yu

**Affiliations:** 1 School of Mechanical and Electrical Engineering, Henan University of Science and Technology, Luoyang 471003, China; zeh@stu.haust.edu.cn (E.Z.); wangjh@haust.edu.cn (J.W.); yxj20061005@163.com (X.Y.); 2 School of International Education, Henan University of Science and Technology, Luoyang 471003, China

**Keywords:** laser cladding, gas–solid two-phase flow, powder convergence characteristics, CFD-DEM

## Abstract

Laser cladding processing efficiency is often limited by low powder utilization. To address this, our study elucidates the mechanism by which powder feeding parameters influence powder stream convergence, aiming to optimize these parameters. A three-dimensional model of a wide-band symmetrical nozzle was developed using a Computational Fluid Dynamics—Discrete Element Method (CFD-DEM) coupling method to simulate the gas–solid flow. Single-factor tests and experimental validation confirmed the model’s reliability. The results identify carrier gas flow as the key parameter controlling the focal length and powder concentration, while the powder feed rate primarily governs the concentration on the focal plane. These findings provide a theoretical foundation for optimizing laser cladding parameters to enhance powder utilization.

## 1. Introduction

With the characteristics of high precision and low heat affected zone, laser cladding technology has significant advantages in the fields of aero-engine blade repair and automobile mold strengthening [1,2]. However, the quality of the cladding layer depends on the stability of the powder conveying process [3,4]. At present, the main defects of laser cladding are focused on the poor stability of powder conveying and the low utilization rate of powder [5,6]. The existing research is mostly based on experimental research. This limitation leads to the fact that the optimization of powder feeding process parameters depends on empirical trial and error, which seriously restricts the cladding efficiency [7,8]. Therefore, using the CFD-DEM method to study the flow field of the wide-band powder feeding nozzle has important guiding significance for optimizing the nozzle structure, improving the cladding efficiency and optimizing the powder feeding process parameter [9,10,11].

Regarding this key fluid dynamics issue, the existing research mainly focuses on coaxial powder feeding nozzles and generally employs computational fluid dynamics methods. For instance, Wang et al. [12] used CFD simulation to reveal the dynamic evolution and particle trajectories of the powder flow beneath the nozzle; Su et al. [13] combined response surface method and neural network to optimize the nozzle structure parameters to improve powder convergence. In terms of simulation methods, Lu [14] and Zhou et al. [15] both used Fluent software to respectively explore the influence of carrier gas flow rate on the conveying stability and the role of geometric shape on the convergence characteristics. Additionally, Yang et al. [16] studied the improvement effect of particle collision momentum loss on convergence, Jin et al. [17] established a powder flow field model based on Euler–Lagrange method, Zhao et al. [18] analyzed the influence of structural parameters on the spot size of broadband coaxial powder feeding nozzles, and Yin et al. [19] simulated the four-channel nozzle and investigated the influence of elastic recovery coefficient on the flow. Despite these explorations, the existing research still has significant limitations: firstly, most of the work focuses on traditional coaxial nozzles. For the broadband symmetric powder feeding nozzle, which is commonly used for large-area cladding, the systematic analysis of the internal and external flow fields is still relatively lacking. Secondly, the research mostly focuses on the influence of a single structural parameter or other particle parameters, and the internal mechanism of how the powder feeding rate and carrier gas flow rate these two core process parameters jointly determine that the powder aggregation characteristics have not been clearly clarified. Finally, most simulations use the continuum assumption to handle the powder phase, making it difficult to accurately capture the real impact of collisions, friction, and other factors at the particle scale on the aggregation behavior.

Compared to conventional coaxial nozzles, the wide-band symmetrical nozzle employed in this study offers inherent advantages for large-area cladding due to its rectangular outlet geometry, which enables a wider single-track deposition width and reduces the number of overlapping passes required to cover a given surface. However, this specific geometric structure leads to complex lateral diffusion and uneven particle concentration distribution, which cannot be adequately simulated by simplified two-dimensional or continuous models. To address this, the present work utilizes a CFD-DEM bidirectional coupling approach. Unlike pure CFD simulations that treat the powder phase as a continuous fluid—thereby neglecting particle-scale interactions—the CFD-DEM framework explicitly resolves inter-particle collisions, wall friction, and momentum exchange at the individual particle level. This is particularly critical for the wide-band nozzle, where particle–particle and particle–wall collisions within the narrow rectangular channels significantly influence the final convergence behavior.

By adopting this method, this paper established a three-dimensional numerical model of a wide-band symmetrical powder conveying nozzle based on the CFD-DEM- combined approach. This model can accurately depict the bidirectional coupling effect between gas turbulence and discrete particles, aiming to systematically clarify the influence laws and mechanisms of powder feeding parameters on the convergence characteristics of the powder flow. The model results will be verified through experiments, and it is expected to provide reliable theoretical basis and design framework for optimizing laser deposition process parameters and designing efficient powder conveying nozzles.

## 2. Materials and Methods

### Basic Principles of Laser Cladding Technology

**Figure 1:**
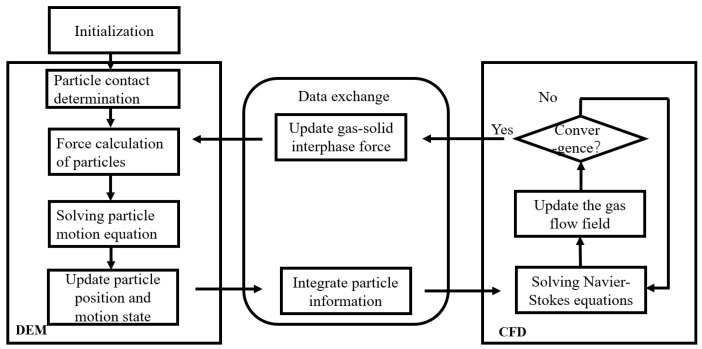
Schematic diagram of the process framework of the CFD—DEM-coupled computing model.

The calculation model is shown in Figure 1 [20,21].

Continuity equation and momentum conservation equation [22]:

(1) $$\frac{\partial }{\partial t}(\alpha \rho )+\frac{\partial }{\partial x_{i}}(\alpha \rho u_{i})=0$$

(2) $$\frac{\partial }{\partial t}(\alpha \rho u_{i})+\frac{\partial }{\partial x_{j}}(\alpha \rho u_{i}u_{j})=-\frac{\partial p}{\partial x_{i}}+\frac{\partial \tau_{ij}}{\partial x_{j}}+\alpha \rho g+S_{q}$$

(3) $$\alpha =1-\sum_{i=1}^{n}\frac{V_{p,i}}{\Delta V}$$

(4) $$\tau_{ij}=(\mu +\mu_{t})\left(\frac{\partial u_{i}}{\partial x_{j}}+\frac{\partial u_{j}}{\partial x_{i}}\right)-\frac{2}{3}\rho k\delta_{ij}$$

(5) $$\mu_{t}=\rho C_{\mu }\frac{k^{2}}{\varepsilon }$$

(6) $$S_{q}=\frac{\sum_{i=1}^{n}F_{d}}{\Delta V}$$

In the equation: *i* and *j* are the directions of the coordinate vectors, *t* is time, *x* is the coordinate, *ρ* is the gas density, *α* is the porosity near the particles, *u_i_* and *u_j_* are the gas velocities in the *i* and *j* directions, *P* is the gas pressure, *S_q_* is the source term of the interaction between gas and solid phases, and *τ_ij_* is the stress tensor. *V_p_*_,_*_i_* represents the volume of particle *i*, Δ*V* is the volume of the selected computational fluid dynamics unit, *μ* is the gas viscosity coefficient, *μ_t_* is the turbulent viscosity coefficient, *δ_ij_* is the Kronecker number, and it is 0 when *i* and *j* are equal.

(7) $$\frac{\partial }{\partial t}(\rho k)+\frac{\partial }{\partial x_{i}}(\rho ku_{i})=\frac{\partial }{\partial x_{j}}\left[\left(\mu +\frac{\mu_{t}}{\sigma_{k}}\right)\frac{\partial k}{\partial x_{j}}\right]+G_{k}+G_{b}-\rho \varepsilon$$

(8) $$\frac{\partial }{\partial t}(\rho \varepsilon )+\frac{\partial }{\partial x_{i}}(\rho \varepsilon u_{i})=\frac{\partial }{\partial x_{j}}\left[\left(\mu +\frac{\mu_{t}}{\sigma_{\varepsilon }}\right)\frac{\partial \varepsilon }{\partial x_{j}}\right]+C_{1\varepsilon }\frac{\varepsilon }{k}(G_{k}+C_{3\varepsilon }G_{b})-C_{2\varepsilon }\rho \frac{\varepsilon^{2}}{k}$$

(9) $$G_{k}=\mu_{t}\left(\frac{\partial u_{i}}{\partial x_{j}}+\frac{\partial u_{j}}{\partial x_{i}}\right)\frac{\partial u_{j}}{\partial x_{j}}$$

(10) $$G_{b}=-g_{i}\frac{\mu_{t}}{\rho P_{\mathrm{rt}}}\frac{\partial \rho }{\partial x_{j}}$$

In the above equation: *C_μ_* takes the empirical coefficient of 0.09, *k* represents the turbulent kinetic energy, *ε* is the dissipation rate, *P*_rt_ is the Prandtl number of turbulence energy, *g_i_* is the component of gravity in the *i* direction, *G_k_* is the turbulent kinetic energy generated by the average velocity gradient, *G_b_* is the turbulent kinetic energy generated by buoyancy, *σ_k_*, *σ_ε_*, *C*_1_*_ε_*, *C*_2_*_ε_*, *C*_3_*_ε_* are empirical constants, with values of 1.0, 1.33, 1.44, 1.92, and 1.2 respectively.

The governing equations for the translation and rotation of particles are respectively expressed as [23]:

(11) $$m_{p,i}\frac{du_{p,i}}{dt}=G_{p,i}+F_{d,i}+\sum_{j=1}^{k_{i}}\left(F_{n,ij}+F_{t,ij}\right)$$

(12) $$I_{i}\frac{d\omega_{p,i}}{dt}=\sum_{j=1}^{k_{i}}\left(F_{t,ij}\times R_{ij}-M_{ij}\right)$$

In the equation, *m_p_* represents the mass of the particle, *u* is the velocity vector of the continuous phase fluid, *u_p_* is the velocity vector of the discrete phase particles, *ρ* is the density of the continuous phase fluid, *ρ_p_* is the density of the discrete phase particles, *I_i_* is the moment of inertia of the particle, ω*_p,i_* is the angular velocity of the particle, *k_i_* represents the total number of particles that particle *i* is in contact with, *R_ij_* represents the vector from the center of mass of particle *i* to the center of mass of particle *j*, *F_n_*_,_*_ij_* and *F_t_*_,_*_ij_* are the normal and tangential contact forces of the particle, and *M_ij_* is the rolling resistance moment between particle *i* and particle *j*.

**Figure 2:**
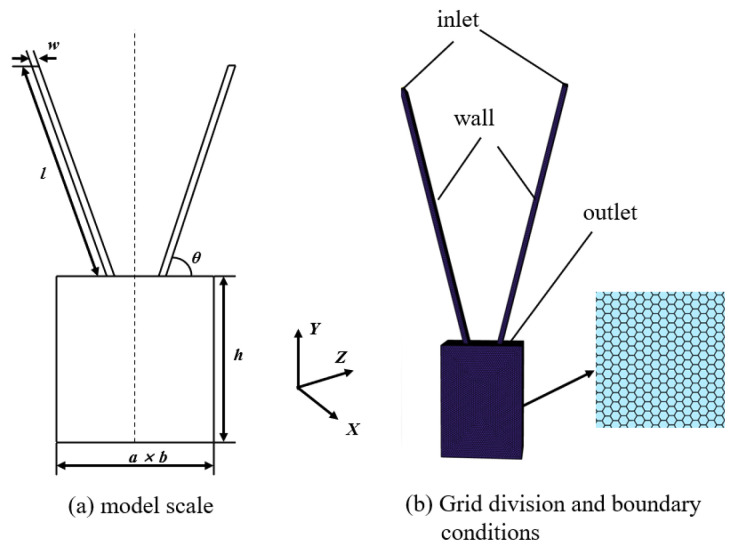
Physical model of the rectangular nozzle and its boundary conditions.

The simulation uses a bidirectional coupling of Fluent and EDEM. Fluent calculates the gas phase flow field, and EDEM calculates the particle phase. After the simulation post-processing, the focal length was measured by Tools-Ruler in EDEM, while in the experiment, the corresponding focal length and divergence angle were measured by ImageJ. The model is shown in Figure 2a. The inlet mesh is locally refined, and the mesh size is refined to 0.3 mm. The volume mesh is divided by polyhedra honeycomb mesh, and the mesh size is 0.6 mm. As shown in Figure 2b. Powder particle parameters are set in EDEM, as shown in Table 1 [23,24], wall parameters and contact types are shown in Table 2 and Table 3 [23,24].

**Figure 3:**
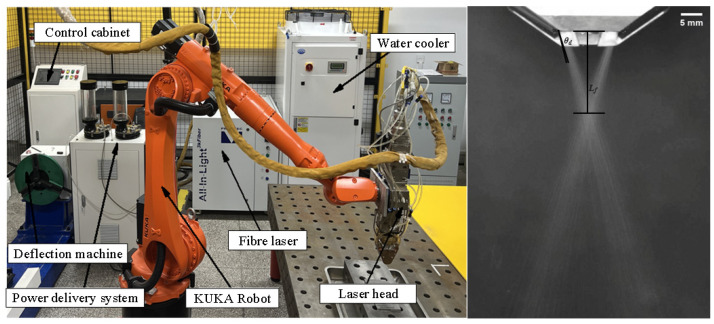
Laser cladding equipment diagram and its evaluation parameter diagram.

The laser cladding equipment uses a KUKA KR 20 R1810-2-type robot, a wide-band laser cladding powder feeding nozzle, and a powder feeding system of the JS-SFQ-02-2-type gas-carrying powder feeder. Argon gas is used as the powder feeding gas. A CCD camera is used to capture the powder flow field characteristics outside the nozzle. The experimental equipment diagram is shown in Figure 3 (left). During the powder feeding process of the powder feeding nozzle of wide-band laser cladding, the gas flow rate drives the powder particles to be ejected from the powder feeding pipe and freely converge. To more intuitively evaluate the powder convergence characteristics and thereby quantitatively characterize its influence, this paper defines evaluation parameters such as powder focal length and powder divergence angle to evaluate the powder convergence. As shown in Figure 3 (right), the divergence angle *θ_d_* is the angle between the axis of the rectangular pipe and the outward divergence of the gas–powder flow after it is ejected from the nozzle. The larger *θ_d_* is, the more dispersed the gas–powder flow is, and the worse the powder convergence characteristics are. The smaller *θ_d_* is, the more concentrated the powder flow is, and thus more powder falls into the molten pool, resulting in higher powder utilization efficiency. The powder focal length *L_f_* is the distance between the intersection point of the powder beam convergence center and the rectangular pipe outlet. The size of the focal length directly determines the size of the light spot and the energy density. By controlling an appropriate focal length, a well-bonded cladding layer can be obtained.

**Figure 4:**
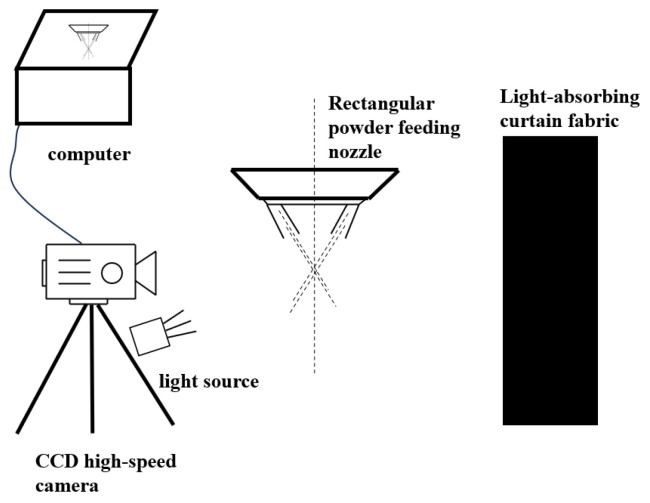
Schematic diagram of the powder beam convergence experiment.

The powder beam convergence experiment is shown in Figure 4. CCD camera is used to simulate and compare the morphological characteristics of the sprayed powder beam to verify the accuracy of the numerical model of the powder beam. The frame number is 200 frames and the resolution is 1280 × 1024. The auxiliary light source is used to light the powder outside the nozzle, and the light-absorbing curtain is placed opposite the CCD camera.

## 3. Results and Discussion

**Figure 5:**
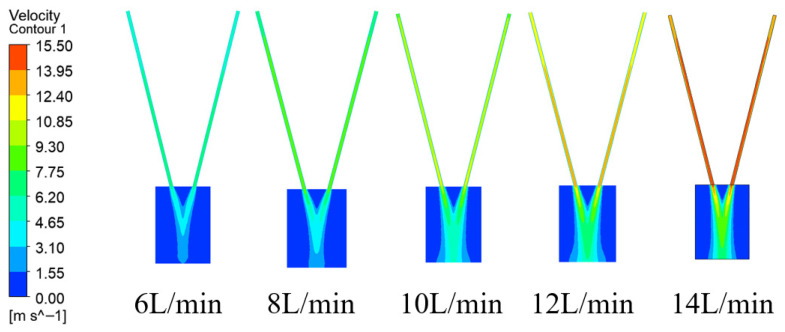
Contours of velocity field distribution at different carrier gas flow.

The primary role of the carrier gas in wide-band laser cladding—transporting powder steadily from the feeder to the molten pool—was examined by studying different flow rates (*Q_g_* = 6, 8, 10, 12, 14 L/min) at a powder feed rate of 33.3 g/min. The gas velocity distributions under these conditions, presented in Figure 5, show a direct correlation between carrier gas flow and increased airflow speed. This accelerated airflow propels the particles through the nozzle’s rectangular pipe, leading to a rapidly diffusing flow field at the outlet.

**Figure 6:**
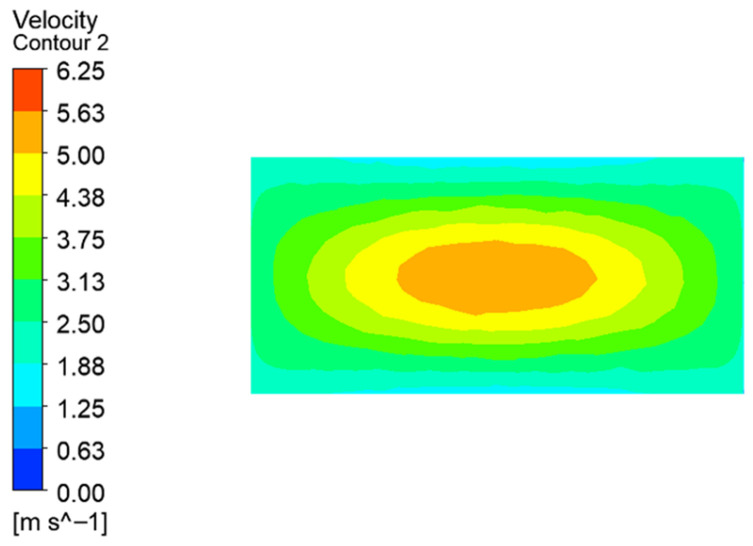
Focus on the distribution of plane velocity Contour map.

Figure 6 shows the distribution of the gas flow velocity in the focal plane, which presents an approximately Gaussian distribution pattern and has good uniformity in flow velocity. This distribution is consistent with the convergence shape of the rectangular light spot, which is conducive to the directional transport of powder particles to the molten pool and can to some extent suppress oxidation during the cladding process.

**Figure 7:**
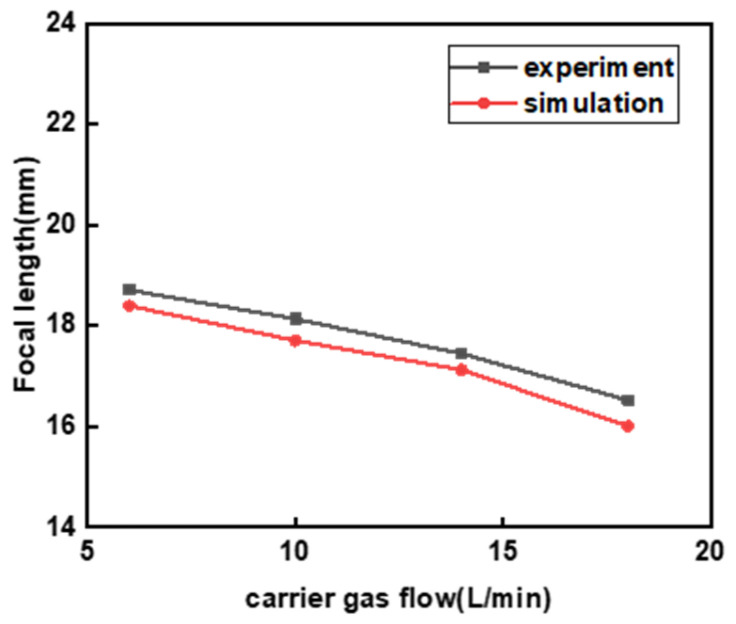
Effect of different carrier gas flow on focal length.

As shown in Figure 7, the simulation results indicate an inverse relationship between the carrier gas flow rate and the focusing characteristics of the powder beam. Specifically, the higher the flow rate, the closer the focusing position is to the nozzle, and the shorter the focusing length. At 6 L/min, the maximum *L_f_* observed was 18.708 mm. At 10 L/min, this value slightly decreased to approximately 18 mm, indicating that the influence is limited within this range. However, at 14 L/min, the *L_f_* significantly decreased to 17.153 mm, and at 18 L/min, it sharply dropped to 15.387 mm. This indicates that an excessively high flow rate significantly alters the focusing position. The underlying mechanism is that the increased gas flow rate increases the gas velocity, which increases the stiffness of the powder beam after it exits the pipe, thereby raising the focus when the powder converges. The maximum relative error between the simulated and experimentally measured focal lengths is 3.2% (observed at 18 L/min), indicating satisfactory consistency and thereby validating the reliability of the numerical model.

**Figure 8:**
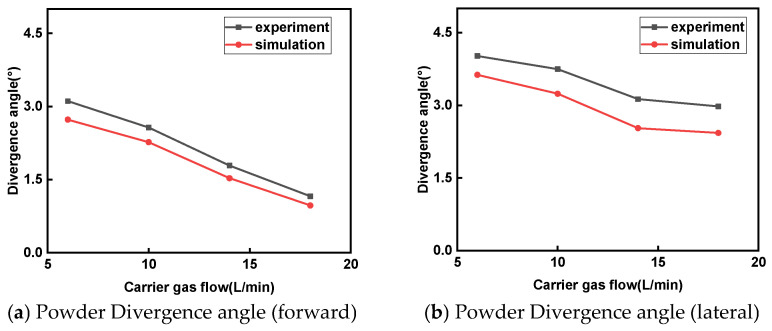
Comparison of simulated and experimental values of the divergence angle of the powder beam.

**Figure 9:**
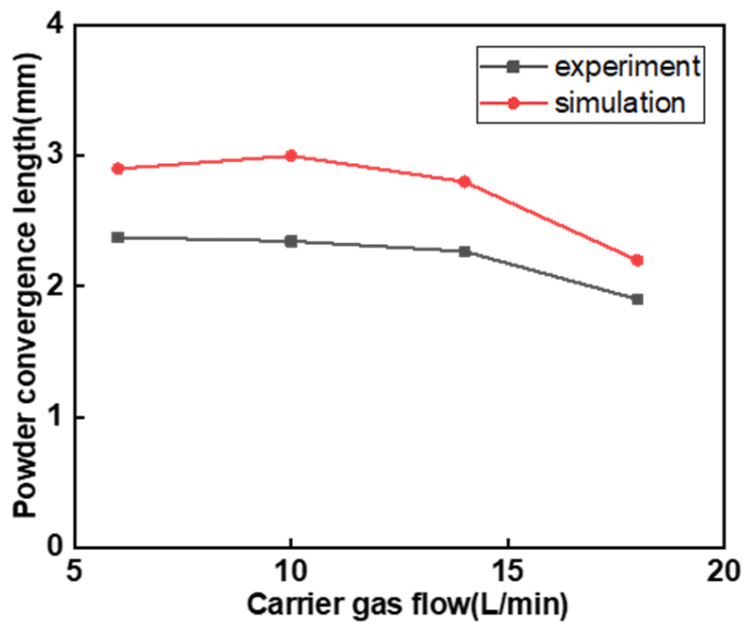
Influence of carrier gas flow on powder convergence length.

**Figure 10:**
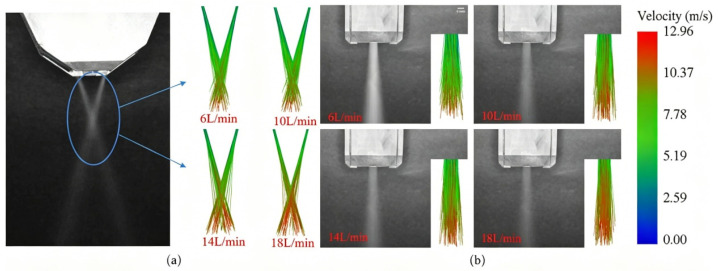
Diagram of the trajectory of the powder beam (**a**) Powder beam trajectory (forward) (**b**) Powder beam trajectory (lateral).

For the wide-band laser cladding powder feeding nozzle, its symmetrical rectangular channel design causes the powder particles to simultaneously undergo forward and lateral divergence at the nozzle outlet. The Figure 10a shows the comparison between the experiment and simulation of the forward divergence angle *θ_d_*_1_, and the Figure 10b shows the comparison between the experiment and simulation of the lateral divergence angle *θ_d_*_2_. As shown in Figure 8, as the carrier gas flow rate increases, the forward *θ_d_*_1_ decreases from 2.73° at 6 L/min to 0.97° at 18 L/min. When the carrier gas flow rate increases from 6 L/min to 18 L/min, the lateral *θ_d_*_2_ decreases from 3.63° to 2.43°, indicating that the *θ_d_* gradually decreases with the increase in *Q_g_*. The reduction in *θ_d_* also results in a slight decrease in the powder convergence length, as shown in Figure 9. As the carrier gas flow rate increases, the powder beam still maintains a high rigidity, as shown in Figure 10, which presents the divergent trajectories of the powder beam in the horizontal and lateral directions. The left side shows the convergence of the powder beam during the experiment, and the right side shows the movement trajectory of the powder beam in EDEM. From the figure, it can be seen that as the carrier gas flow rate increases, the divergence angle of the powder beam decreases to a certain extent, but from the simulation images, it can be seen that the speed of the particles is constantly increasing, and the color of the trajectory represents the speed of the powder particles. However, an excessively high carrier gas flow rate will cause the powder to splash on the substrate, thereby reducing the deposition efficiency. Therefore, appropriately increasing the carrier gas flow rate is helpful in improving the convergence characteristics of the powder beam.

**Figure 11:**
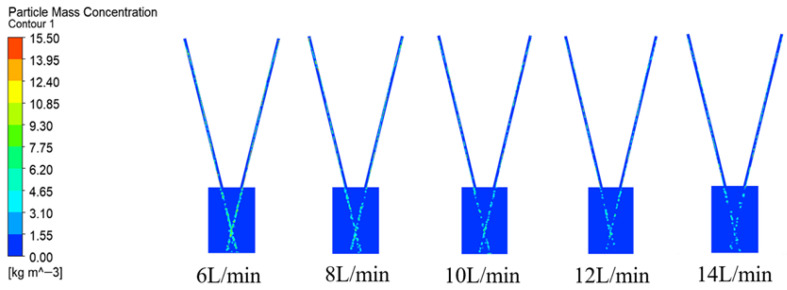
Contours of powder mass concentration distribution at different carrier gas flow.

Figure 11 shows the particle mass concentration distribution Cp on the XOY plane. As the carrier gas flow increases, when it reaches 14 L/min, due to the excessive flow, the gas flow moves the powder particles at a faster speed, and the powder particles are easily affected by the gas flow disturbance. As a result, the diffusion within the calculation domain is severe, and it is difficult to achieve the ideal convergence effect within the focused range, leading to poor powder convergence.

**Figure 12:**
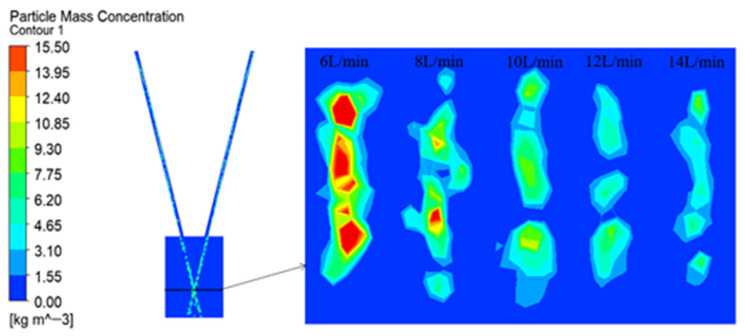
The concentration distribution contour map of powder particles on the focal plane.

Figure 12 shows the concentration distribution cloud map of the powder particles on the focal plane. The overall convergence trend of each figure conforms to the convergence effect of the symmetrical rectangular channel powder feeding nozzle. It presents a rectangular distribution. At 6 L/min, the influence of the airflow disturbance is relatively weak, and the distribution is relatively uniform. As the carrier gas strength increases continuously, the turbulent effect of the airflow becomes more obvious, and the airflow disturbance on the convergence plane becomes stronger, resulting in a more significant decrease in the mass concentration of the particles.

**Figure 13:**
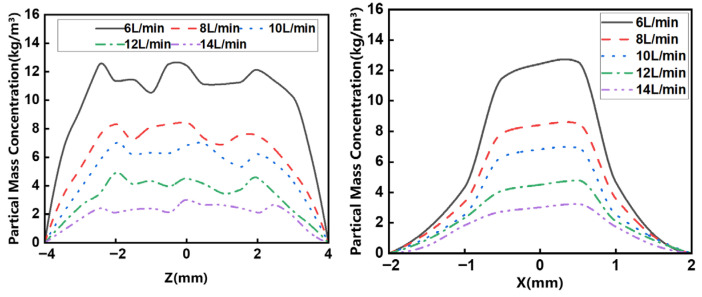
Distribution of powder mass concentration at different carrier gas flow rate.

Figure 13 illustrates the powder concentration profile resulting from the unique symmetrical design of the wide-band nozzle, characterized by fluctuations in the z-direction and a near-Gaussian distribution in the x-direction on the focal plane. The optimal powder convergence is achieved at 6 L/min. The Particle Mass Concentration reached 12.55 kg/m^3^. Moreover, higher carrier gas flows increase particle velocity. With the powder feed rate fixed, this elevated velocity leads to a pronounced reduction in powder concentration.

**Figure 14:**
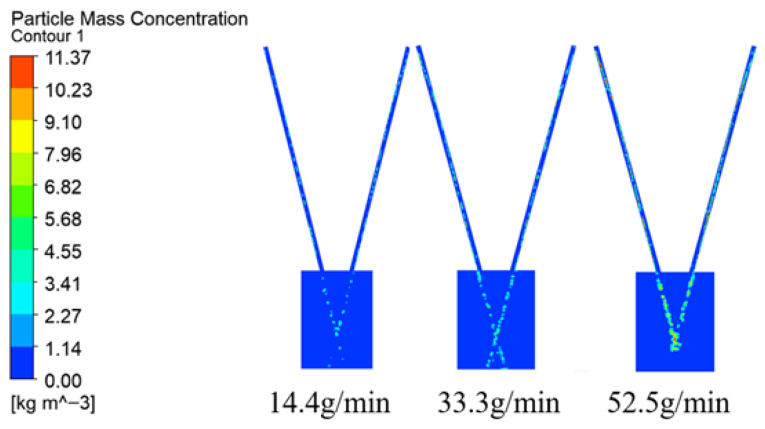
Contours of powder mass concentration at different power feeding rate.

**Figure 15:**
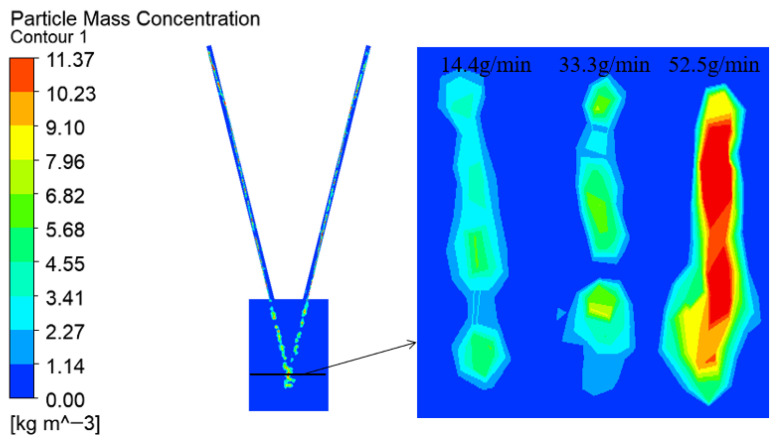
Contours of concentration distribution on the focal plane with different power feeding rate.

The effect of powder feeding rate on convergence was investigated with a fixed carrier gas flow. The results indicate a direct relationship: a higher feeding rate leads to a greater powder mass concentration (Figure 14). This correlation is due to the increased mass of powder transported within the same duration, which elevates particle density in the convergence area. Consequently, the focal plane concentration contours (Figure 15) consistently display a rectangular distribution, matching the laser spot’s geometry.

**Figure 16:**
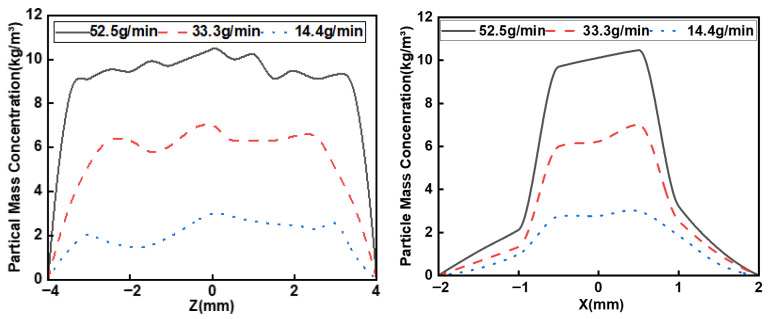
Distribution of powder mass concentration at different power feeding rate.

The powder mass concentration, depicted in Figure 16, follows the carrier gas flow distribution in both the x and z directions. This concentration is also positively correlated with the powder feeding rate, evidenced by its rise from 2.98 kg/m^3^ to 10.47 kg/m^3^ as the rate increased from 14.4 g/min to 52.5 g/min.

To investigate the influence of the physical properties of the powders on the transportation and convergence processes, this study selected three types of metal powders that are widely used in laser cladding: 17-4PH stainless steel, 316 L stainless steel, and Inconel 718 nickel-based alloy. Under fixed process parameters (gas flow rate of 10 L/min, powder feeding rate of 33.3 g/min), the flow field characteristics of these powders were compared and analyzed.

**Figure 17:**
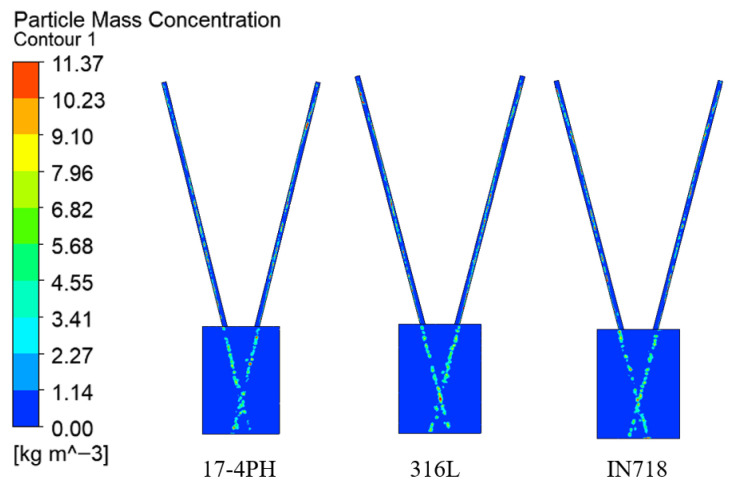
Contour map of powder mass concentration under different powder materials.

**Figure 18:**
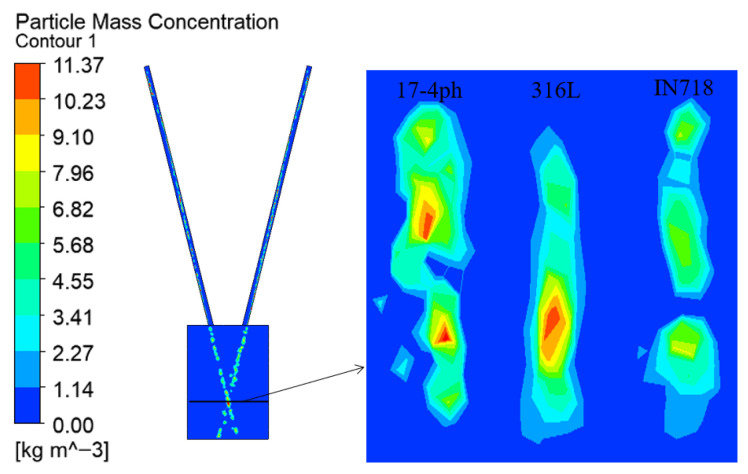
The concentration distribution contour map of the focal plane under different powder materials.

Figure 17 and Figure 18 demonstrate that the concentration distribution maps of the three powders all exhibit characteristics similar to those of rectangular powder spots. The concentrations of the three powders are basically the same whether considering the concentration distribution during transportation to the convergence area or the specific concentration distribution on the focal plane. This is because under the same powder feeding rate and carrier gas flow rate, although the volume of powder delivered to the convergence area varies in units of time, the spatial diffusion behavior patterns after passing through the gas flow field are basically the same, ultimately resulting in a very slight difference in the calculated mass-concentration field.

The observed trends can be elucidated through the lens of gas–solid momentum coupling. In a dilute phase flow regime, particle inertia is predominantly governed by the drag force exerted by the carrier gas. As the carrier gas flow rate increases, the higher gas velocity imparts greater axial momentum to the particles, effectively increasing the stiffness of the powder stream. This enhanced momentum suppresses the initial expansion driven by turbulent diffusion at the nozzle exit, thereby reducing the divergence angle. Furthermore, the high-velocity jet creates a low-pressure entrainment zone which forces the streams to converge at a closer distance, explaining the observed reduction in focal length. However, excessive gas flow leads to particle splashing upon substrate impact, offsetting the benefits of improved convergence. Conversely, the powder feed rate primarily modulates the particle number density within the flow field. Given the low volume fraction of the solid phase, It has a relatively small impact on the overall flow trajectory of the fluid. Consequently, the focal length remains invariant, while the mass concentration on the focal plane exhibits a near-linear correlation with the powder feeding rate.

## 4. Conclusions

In this study, numerical simulation and experimental verification were conducted on the powder distribution pattern and convergence characteristics of the external flow field of a broadband symmetrical rectangular powder feeding nozzle by coupling Fluent 2022 with EDEM 2023 software. The results show that the simulation model can accurately predict the dynamic behavior of the powder beam under different feeding process parameters, and the consistency with the experimental data verifies the reliability of the model. The findings are as follows:

(1) The focal length and powder mass concentration of the powder beam decrease continuously as the carrier gas flow Qg increases. During the process of increasing the carrier gas flow from 6 L/min to 18 L/min, the focal length of the powder beam gradually decreases from 18.708 mm at 6 L/min to 18 mm at 10 L/min, and then drops sharply to 15.387 mm at 18 L/min. The peak concentration of the powder beam at the convergence point of the powder mass concentration decreases from 12.43 kg/m^3^ at 6 L/min to 3.1 kg/m^3^ at 14 L/min. An appropriate increase in the carrier gas flow can improve the convergence characteristics of the powder flow and thereby reduce the divergence angle of the powder flow.
(2) The powder mass concentration increases continuously from 2.98 kg/m^3^ at 14.4 g/min to 10.47 kg/m^3^ at 52.5 g/min. The effect of the powder feeding rate mp on the external flow field convergence characteristics of the powder beam is not significant but has a significant impact on the powder mass concentration.

## Figures and Tables

**Table 1:** Parameters Related to Powder Particles.

Particle Type	Parameters	Value
Inconel718	Density (kg/m^3^)	8240
	Shear modulus (Pa)	7.72 × 10^10^
	Poisson’s ratio	0.3

**Table 2:** Wall surface-related parameters.

Wall Type	Parameters	Value
Cu	Density (kg/m^3^)	8920
	Shear modulus (Pa)	4.8 × 10^10^
	Poisson’s ratio	0.33

**Table 3:** Contact model-related parameters.

Type	Parameters	Value
Particles and particles	Collision recovery coefficient	0.5
	Coefficient of static friction	0.5
	Coefficient of kinetic friction	0.01
Particles and walls	Collision recovery coefficient	0.3
	Coefficient of static friction	0.4
	Coefficient of kinetic friction	0.01
	Contact model	Hertz-mindlin (no slip)

## Data Availability

The original contributions presented in the study are included in the article further inquiries can be directed to the corresponding author.
